# Utilization of growth monitoring and promotion is highest among children aged 0–11 months: a survey among mother-child pairs from rural northern Ghana

**DOI:** 10.1186/s12889-021-10980-w

**Published:** 2021-05-13

**Authors:** Fusheini Seidu, Victor Mogre, Adadow Yidana, Juventus B. Ziem

**Affiliations:** 1 Department of Community Health and Family Medicine, School of Medicine and Health Sciences, University for Development Studies, Tamale, Ghana; 2 Department of Health Professions Education and Innovative Learning, School of Medicine and Health Sciences, University for Development Studies, P. O. Box TL 1883, Tamale, Ghana; 3 Department of Clinical Microbiology, School of Medicine and Health Sciences, University for Development Studies, Tamale, Ghana

**Keywords:** Growth monitoring and promotion, Utilization, Child health, Nutrition surveillance, Mothers, Ghana

## Abstract

**Background:**

More than half of all deaths in under 5 children is related to malnutrition. Child malnutrition could be prevented through regular monitoring of the growth and development of children and the implementation of growth promotion activities referred to as growth monitoring and promotion (GMP). Mothers’/caregivers utilization of these activities through child welfare clinics could improve the growth and development of under 5 children. We evaluated mothers’ knowledge on GMP, utilization and associated factors among mother-child pairs from a poor socio-economic district in Northern Ghana.

**Methods:**

Using an analytical cross-sectional design, participants included mothers with children aged 0–59 months, grouped into 0–11 months, 12–23 months and 24–59 months. A semi-structured questionnaire containing both closed- and open-ended questions was used to collect data. Multivariate logistic regression was used to identify determinants of GMP utilization.

**Results:**

Four hundred mother-child pairs were included in the study. Overall, 28.5% (*n* = 114) of the mothers utilized GMP services. Almost 60%(*n* = 237) of the mothers knew the recommended age to seek for GMP service for their children. Only 9% of the mothers could correctly interpret the directions of the growth curves in their children’s Health Record booklet. Mothers with children aged 0–11 months were 3.9 times more likely (*p* = 0.009) to utilize GMP services compared to their counterparts with children aged 12–23 months and 24–59 months. Mothers who had low level of knowledge were 2.19 times (*p* = 0.003) more likely to utilize GMP services compared to their counterparts with high level of knowledge..

**Conclusion:**

Utilization of GMP services was low and particularly lower in children aged 24–59 months. Mothers’ knowledge in GMP was optimal although there were notable gaps.

## Background

Child malnutrition is a universal public health issue faltering development and causing undesirable consequences to human health [1]. Over half of all deaths in under 5 children is linked to malnutrition as it places children at increased risk of death from common infections, increases the episodes and intensity of such infections and prolongs recovery [2] . In 2019, 144 million under 5 children in the world were stunted, 47 million wasted of which 14.3 million were severely wasted and 38.3 million overweight [3]. In sub-Sharan Africa, 57.5 million under 5 children in 2019 were stunted, rising from 49.2 million in 2000; 11.8 million wasted in which 3.0 million had severe wasting and 5.2 million were overweight. In Ghana, 19% of under five children were stunted, 11% underweight and 5% wasted in 2014 [4]. The Northern Region of Ghana has the highest rate of stunted children with a prevalence of 33.0% [4].

One of the strategies that have been introduced in developing countries and other parts of the world towards reducing malnutrition in children is growth monitoring and promotion (GMP). GMP refers to a nutritional intervention in under 5 children that regularly measures and plot the weight of children less than 5 years in a growth graph within specific intervals as well as comparing the growth curves to that of a reference population and the results used to counsel caregivers to enable them take concrete steps that will improve the growth of the child [5–7]. It offers an early warning signal for appropriate and timely action to be taken and therefore identifies and correct growth faltering before a child’s nutritional status degenerates into a fully blown malnourished state [6–8]. In addition, GMP facilitates engagement and communication with caregivers thereby further providing the necessary action that is aimed at promoting child growth and increasing demand for other essential services [6, 8].

Implemented in the 1970’s by UNICEF, the GMP is a component of the Child Welfare Clinic (CWC) package in Ghana [9]. The CWC is a comprehensive child health care package that involves the provision of immunization, food and/or micronutrient supplementation, growth monitoring, nutrition education and counselling [10]. These services are provided during monthly visits to the health facility. GMP has been shown to have the capacity to maintain and improve child feeding and care practices [11]. Notwithstanding these benefits the implementation of GMP is frequently limited by ineffectiveness in the conduct of the promotion aspect of GMP (i.e. nutrition education and counselling to improve child nutrition) as healthcare providers usually focus on growth monitoring which on its own does not really result in improving the nutritional status of children [11].

Rates of utilization or attendance of GMP sessions in sub-Sharan Africa and in other developing countries has been reported to be varied [11]. In Senegal, 70% of children attended monthly GMP provided by a non-governmental organization, whereas 35% patronized the government facility [11]. Another study in Ethiopia among children aged 0–24 months revealed a GMP utilization rate of 16.9% [12]. In the Ga West District of Ghana, Agbozo et al [10] reported 13.6% of caregiver-child pairs obtaining more than 9 visits in the last 12 months. Gyampo et al reported 70% of mothers not missing scheduled child welfare clinic session in a study conducted in the Accra Metropolitan Area in Ghana [13].

Notwithstanding the fact that GMP has been implemented in Ghana since the 1970s, evidence regarding the utilization of GMP services and its associated factors among mothers with their children is limited. In a review of the literature we came across three studies [10, 13, 14] in which only one study was conducted in the Northern part of Ghana [14] but the rest were conducted in Accra that has a higher socio-economic status compared to Northern Ghana. The study conducted in Northern Ghana focused on the knowledge of health workers and a couple of mothers/care givers but did not measure utilization of GMP services [14]. In addition, the study was limited by a small sample size of 78 health workers and 35 mothers/care givers. Also, evidence regarding the association between mothers’ knowledge on GMP and its utilization is scanty. The current study intends to fill these gaps in the literature from a poor socio-economic setting of the Nanumba South District of Ghana where we investigated the prevalence of utilization of GMP. In addition, the association between mothers’ knowledge on GMP and its utilization was evaluated. The study will provide context-based, local evidence to inform policy at the district, regional and probably national levels towards the development and implementation of strategies to improve the utilization of GMP by mothers/care givers for children less than 5 years. This evidence will also be relevant to countries with similar economic and developmental settings like Northern Ghana.

## Methods

### Study design and setting

This cross-sectional study was conducted from December 2018 to July 2019 in the Nanumba South district of the Northern Region of Ghana. The Nanumba South District is located about 211 km Southeast of the Northern Regional Capital, Tamale. The district lies within Latitudes 8.5^0^ and 9.0^0^ North and longitudes 0.5^o^ east and 0.5^o^ West of the Greenwich Meridian. It has Wulensi as the administrative capital. According to the 2010 Ghana Population and Housing census, the district has a total population of 114,173 inhabitants at an annual growth rate of 2.9%. The sub-categorical operational populations for 2017 were 4567 for children aged 0–11 months; 4567 for children aged 12–23 months; and 13,472 for children aged 24–59 months. The district has four (4) health centres and eleven (11) functional Community-Based Health Planning and Services (CHPS) compounds across all of its four sub-districts to cater for the health needs of the people living in 137 communities. The average distance to a health facility in the district is about 8 km. Antenatal and child welfare services are organized across 128 static and outreach points where GMP services are provided.

### Study participants, sample size determination and recruitment procedures

The participants of this study were mothers with their children aged 0–59 months (referred to as mother-child pairs) attending monthly child welfare clinics at all the four health centres and one CHPS zone selected from each of the sub-district by way of the lottery method. Using Cochran’s formula n = Z2pq /e2 where n is the sample size, e is the desired level of precision (margin of error), p is the (estimated) proportion of the population which has the attribute in question (GMP utilization rate) or the degree of variability, q is 1-p and Z is the abscissa of the normal curve that cuts off an area at the tails, a sample size of 405 was obtained. A 5% non-response rate was computed to yield a final sample size of 425. Before commencing data collection, written permission was obtained from the health authority at the district, sub-district and facility levels. Each time, a selected health facility was visited, all mother-child pairs that attended the welfare clinics were approached. During this process, the study rationale and procedure were explained to the participants and only those who agreed and provided informed consent were included into the study. This process was continued until the quota allocated for the specific health facility was achieved. Voluntary participation was encouraged throughout the study. Each participant was free to decline at any point of the study without condition. Access and provision of child welfare services to those who agreed to participate in our study as well as those who declined was without any discrimination. Ethical approval was granted by the Ethical Review Committee of the Tamale Teaching Hospital in Ghana.

### Data collection methods

A semi-structured questionnaire was used to collect data. The questionnaire consisted of three sections. Section one evaluated the socio-demographic characteristics of both the mother and the child (i.e., child’s age, child’s gender, mothers’ level of education, income level, marital status, employment status). Child’s age was recorded in months and was obtained by recording the date of birth from the Child Welfare Book. Mothers were asked to indicate their current level of formal education received: No formal education, primary, Junior high school, Senior High School, and Tertiary. The age of the mother was assessed asking the question: How old are you? Income level was evaluated by asking mothers to estimate how much they earned monthly. Mothers were asked to indicate whether they were single, married, cohabiting, widowed or divorced. Employment status was assessed by the asking the question: Which of the following sectors are you currently working: Formal sector, private sector, farmer, trader, and housewife. Section two consisted of both closed and open-ended items that assessed mothers’ knowledge regarding: the purpose of GMP, recommended frequency of attendance of GMP sessions and the use and interpretation of growth charts. The items were derived from previous studies. Participants were given a score of 1 for every correct answer. A total score was generated and computed 100%. Section three assessed the level of GMP utilization by asking the mother and cross-checking in the child health record book the number of times mothers visited the GMP sessions with their children so far, and the number of times they missed GMP sessions. Missed GMP sessions was determined by the difference between the number of visits and the expected number of visits to GMP sessions. Utilization was then determined by the number of visits to the GMP session as per the recommendation in the Child Health Record booklet, which requires monthly visits from aged 0 to 23 months, and a visit at every four months from aged 24 to 59 months (Ghana Health Service, n.d.). To ensure comprehensibility, the questionnaire was pretested among 20 participants selected from a non-participating community within the area having similar characteristics. This allowed for further clarification and modification of some of the items of the question. In addition, the questionnaire was evaluated for content validity by a team of nutritionists, behavioural sciences and public health specialists. Since most of the participants could not read nor write in English, participants were interviewed in their local dialect where necessary. All participants were interviewed in privacy in a secluded area of the facility to ensure confidentiality. All methods were carried out in accordance with relevant guidelines and regulations.

### Statistical analysis

Data was entered onto Microsoft Excel 2010 and analysed using the Statistical Package for the Social Sciences (SPSS) software, version 20. Descriptive statistics of mean and standard deviation was used to describe continuous variables whilst frequencies and percentages were used to describe categorical variables and presented as frequencies and charts. Following the Food and Agriculture Organisation nutrition-related knowledge thresholds for a nutrition intervention in which a knowledge score ≤ 70% shows an urgent need for a nutrition intervention, all mothers who scored > 70% in the knowledge test were classified as having high level of knowledge and those scoring ≤70% were classified as having a low level of knowledge [15, 16]. Quotes from open-ended knowledge questions were used to support the correct answers provided by the mothers. Chi-square test and Fisher’s exact test were used to determine the association among categorical variables where appropriate (i.e., utilization of GMP with socio-demographic characteristics and levels of knowledge). To identify factors associated with the utilization of GMP, multivariate logistic regression was conducted. For the purposes of the analysis the age of the children was categorised into 0–11 months, 12–23 months and 24–59 months. Level of education was categorised into no formal education, low and high levels of education in which senior high school and tertiary levels of education were combined to yield “High level of education” and Primary and Junior High School levels were combined to yield “Low level of education “. Mothers/care givers’ age was categorised into: < 30 years and ≥ 30 years. Based on a median income level of GHC 60, mother’s monthly income level was placed into two categories: ≤ GHC60 (US$ 11) and > GHC 60. Regarding employment status, responses to formal sector, private sector, traders and farmers were categorised into ‘employed’ and those who reported as being housewives was categorised into ‘unemployed’. A *p*-value of less than 0.05 was considered significant in all comparative analysis.

## Results

Four hundred and twenty five participants were approached in which all agreed to participate and were administered questionnaires in which 400 questionnaires were found to be complete and included into the analysis giving a response rate of 94.1%. Table 1 shows the socio-demographic characteristics of the 400 mother-child participants included in the study. The children had a mean (SD) age of 11.36 (8.61) months and 51.0% (*n* = 203) were males. The mothers had a mean (SD) age of 27.29 (5.63) years; 95.3% (*n* = 381) were married and literacy rate among them was 50.8% (*n* = 203). In all, 8.8% (*n* = 35) were educated up to primary school level, 19.0% (76) junior high school level, 20.5% (*n* = 82) high school level and 2.5% (*n* = 10) tertiary level.

**Table 1:** General and socio-demographic characteristics of mother and child

Variable	Frequency	%
**Child’s gender**		
Female	197	49.3
**Child’s age (in months)**		
0–11	228	57.0
12–23	143	36.0
24–59	29	7.0
**Mothers’ age (in years)**		
< 30 years	262	65.5
≥ 30 years	138	34.5
**Mothers’ marital status**		
Married	381	95.0
Not married	19	5.0
**Mothers’ educational level**		
No formal education	197	49.2
Low	111	27.8
High	92	23.0
**Mothers’ employment status**		
Employed	299	74.8
Not employed	101	25.2
**Mothers’ monthly income level (GHC)**		
≤ 60	208	96.8
> 60	185	3.2

Senior high school level and tertiary level were combined to yield “High level of education” and Primary and Junior High School levels were combined to yield “Low level of education”

### Utilization and knowledge level of growth monitoring and promotion (GMP) services

Participants’ mean (SD) number of GMP attendance and missed GMP visits were 7.93 (5.52) and 3.45 (4.50) respectively. Overall, 28.5% (*n* = 114) of the mothers utilized GMP services according to the recommended number of age-specific visits of which, 95.6% (*n* = 109) of them had children aged 0–23 months and for 4.6% (*n* = 5) of them, the children were aged 24–59 months. As shown in Table 2, 59.3% (*n* = 237) of the mothers knew the recommended age to seek for GMP service for their children; and 86% (*n* = 344) knew the recommended number of visits according to the age of the child. This was illuminated by the following quote from one of the participants “*if you deliver for instance today, you begin to send the baby for GMP activities and continue to do so until the child completes all his vaccinations.”* (IDI-7). Another added that *“we send the children for GMP services monthly, when you begin this week, the next visit will be the third week of the following month”* (IDI-4). Regarding the duration of GMP activities, 54% (*n* = 216) of the mothers said GMP visits should be done for the child from birth till 59 months. *“A child is taken to the clinic for weighing until he/she gets up to five years before one can stop”* (IDI-23). Only 37(9%) participants said they could correctly interpret the directions of the growth curves in their children’s Health Record booklet from which all of them correctly indicated the arrow for growth; 27 correctly indicated the arrow for a child that is faltering in weight. In total, participants had a mean (SD) score of 68.61% (9.29). Fifty six percent of the mothers had a high level of knowledge i.e., had a total score > 70%.

**Table 2:** Mothers’ knowledge on the importance of growth monitoring and promotion

Purpose of GMP	Frequency	%
To monitor growth and development of the child	400	100
To prevent child malnutrition	399	99.8
To received vaccinations/immunizations for the child	399	99.8
For the child to receive treatment if sick	334	83.5
To receive education on infant and young child feeding	393	98.3
Knows that recommended time for start of GMP is at birth	237	59.3
Knows the recommended GMP visits for their children	344	86.0
Knows duration of attending GMP is from 0 to 59 months	216	54.0
GMP services are provided at the community level and health centres	394	98.5
Knows the meaning of the growth curve/arrow pointing up	22	5.5
Knows the meaning of direction of curve/arrow neither pointing up or down	15	3.4
Knows the meaning of direction of curve/arrow pointing down	16	4.0
**Classification of knowledge scores**		
High level of knowledge	224	56.0
Low level of knowledge	176	44.0

### Factors associated with utilization of GMP services

As shown in Table 3, the utilization of GMP services by mothers for children aged 0–11 months (88.6%, *n* = 101) was significantly (*p* < 0.001) higher than those aged 12–23 months (7.0%, *n* = 8) and 24–59 months (4.4%, *n* = 5).

**Table 3:** Univariate analysis of factors associated with the Utilization of GMPs services

Variable	Utilizes GMP services		*p*-value
	Yes (***n*** = 114)	No (***n*** = 286)	
**Child’s gender**			0.150
Male	51 (44.7%)	152 (53%)	
Female	63 (55.3%)	134 (47%)	
**Child’s age (months)**			< 0.001
0–11	101 (88.6%)	127 (44.4%)	
12–23	8 (7.0%)	135 (47.2%)	
24–59	5 (4.4%)	24 (8.4%)	
**Mother’s age**			0.130
< 30 years	80 (70.2%)	182 (63.6%)	
≥ 30 years	34 (29.8%)	104 (36.4%)	
**Mothers’ educational level**			0.087
Low/no formal education	81 (71.1%)	227 (79.4%)	
High	33 (22.0%)	59 (20.6%)	
**Mothers marital status**			0.438
Married	107 (94.0%)	274 (95.8%)	
Not married	7 (6.0%)	12 (4.2%)	
M**others’ employment status**			0.899
Employed	86 (75.4%)	213 (74.8%)	
Not employed	28 (24.6%)	73 (25.5%)	
**Mothers’ monthly level of income**			
≤ 60	55 (49.5%)	153 (54.3%)	0.433
> 60	56 (50.5%)	129 (45.7%)	
**Knowledge level**			
Low	59 (51.8%)	117 (40.9%)	0.032
High	55 (48.2%)	169 (59.1%)	

From the multivariate analysis shown in Table 4, mothers with children aged less than 12 months were 3.9 times more likely to utilize GMP services compared to those with children aged 12–23 months (*p* = 0.009).

**Table 4:** Multivariate analysis of factors associated with the utilization of GMP service

Variable	AOR (95% CI)	***p***-value
**Child’s gender**		
Male	0.79 (0.48–1.30)	0.355
Female	1	
**Child’s age**		
0–11 months	3.9 (1.38–10.83)	0.010
12–23 months	0.25 (0.08–0.87)	0.029
24–59 months	1	
**Mothers’ age**		
< 30 years	1.09 (0.61–1.94)	0.767
≥ 30 years	1	
**Educational level**		
No formal education	1	
Low level of education	0.90 (0.48–1.69)	0.750
High level of education	1.51 (0.77–2.94)	0.232
**Marital status**		
Married	0.74 (0.26–2.13)	0.574
Not married/single/divorced/widowed	1	
**Employment status**		
Employed	1.09 (0.58–2.04)	0.787
Not employed		
**Monthly income**		
≥ GHC 60	0.84 (0.49–1.45)	0.526
> GHC 60	1	
**Knowledge level**		
Low	2.19 (1.32–3.64)	0.003
High	1	

1 = Reference value; Cox and Snell R Square = 0.204; Nagelkerke R Square = 0.293

## Discussion

In this study, we evaluated mothers’ knowledge on GMP, utilization of GMP and their associations. We found that mothers had good level of knowledge regarding the purpose of GMP but majority of them did not know how to interpret children’s growth charts. Only 29% of the mothers utilized GMP services (i.e., met the recommended number of age-specific GMP visits) for their children. Knowledge on GMP was not significantly associated with the utilization of GMP services. However, the age of the child was the only factor found to be significantly associated with the utilization of GMP services.

An important finding of this study was that almost all the mothers knew the purpose of GMP i.e., to ensure the growth and development of the child; to prevent the child from becoming malnourished, and for the child to get immunizations/vaccinations. These findings are corroborated by the results of a qualitative study conducted in Ethiopia among health workers and mothers, which revealed that mothers perceived the benefits and purposes of GMP as monitoring the growth and development of the child, reducing child undernutrition, keeping the child healthy and introduction of appropriate child feeding practices [17]. Another study in Northern Ghana [14] reported similar findings.

Children are taken for GMP sessions at birth so that the appropriate feeding and child care practices will be instituted, and also to provide the opportunity for any growth faltering to be detected early and timely actions taken to correct it [11]. However, most of the children in the current study are likely to miss this opportunity considering the fact that 40.7% of the mothers did not know the recommended time to begin attending GMP sessions with their children. The results is higher than the 31.8% that could not tell when to begin sending a child to GMP sessions in a study reported by Daniel et al. [18] among 369 women in Southern Ethiopia.

The WHO recommends that a child should commence GMP sessions from the first month and continue till 59 months [10]., but in the study only 54% of mothers knew of this recommendation. This implies that more than 40% of the mothers are likely to stop attending GMP sessions before their children reach 59 months. This finding is further corroborated by findings of Adu-Gyamfi and Adjei who found 85% of respondents not sending their children to child welfare clinics at ages < 59 months [19]. These findings are worrying as the children may be denied of the other benefits of GMP such as growth monitoring, receiving of vitamin A supplementation and nutrition education and counselling.

We also found in this study that majority of the mothers did not know how to interpret the growth charts of their children. This finding is similar to studies conducted in Southern Ethiopia and Ghana in which 38% of mothers in the Ethiopian study did not know the interpretation and meaning of the growth curve [18] and only 18.7% of the respondents in the Ghanaian study were able to interpret the directions of the growth curves [20]. This is probably due to the fact that the health workers at the child welfare clinics are usually preoccupied with weighing the children and recording in the child health record books and not educating the mothers on it. This is buttressed by findings of a Zambian study where mothers expressed their unhappiness for not being provided any information or advice on the growth monitoring processes by health staffs [21]. Also, a study carried out in the East Mamprusi Municipality of Ghana, by Sulley et al. [14] reported that mothers were of the opinion that health workers do not discuss with them anything concerning the growth and development of their children after weighing sessions and that they were not asked to interpret the growth chart either. This is worrying as it has the tendency of discouraging the mothers from partaking in GMP sessions since they may not appreciate the essence of the service. It also implies that the mothers will not be able to determine if growth faltering had occurred, so as to be prompted to take an action. Health workers should pay more attention to mothers and their children by engaging and discussing with them the growth pattern of the children during weighing sessions using the growth curves in the child health record booklet so as to increase the mothers knowledge and interest in GMP activities.

Another important finding of this study was that only 28.5% of the mothers utilized GMP according to the age-specific number of times recommended by the WHO. The finding is slightly higher than the 26% reported in a 3 months cohort study in Zambia which evaluated the effectiveness of institutional based GMP [21]. It is however lower than the 67% reported in South Africa in an assessment of a 5 year GMP project among caregivers with children less than 5 years [22]. The differences may be due to the fact that the GMP activity in South Africa was a project in which a lot of efforts and strategies might have been put in place to motivate caregivers to attend.

An important finding of this study was that we found that age was significantly associated with utilization of GMP services by the mothers in that mothers with children aged 0–11 months were 3.9 times more likely to utilize GMP services compared to those aged 12–23 months and 24–59 months. The finding is consistent to the study by Owusu and Lartey [23] who found that there was a negative correlation between the age of the child and attendance to child health clinics. It is also corroborated by the finding in a mixed method study conducted in Ghana that found 70% of the study participants stopped sending their children to child welfare clinics when the children were within the age group of 12–23 months [19]. This trend may be attributed to the value mothers placed on vaccination over other services of the GMP sessions. Children within the age group of 0–11 months are recommended to go for several doses of immunization and so most mothers will continue to send them to weighing centres until completion of the immunization. In that as the child advances in age, attendance to child welfare clinic decreases. The result is further consolidating the general view that growth monitoring and promotion service utilization is poor among children especially those in the aged group of 24–59 months.

We also found that mothers’ knowledge regarding GMP was reversely associated with the utilization of GMP services in that those who had high level of knowledge were less likely to utilize GMP services compared to their counterparts who had low level of knowledge. This implies that acquisition of knowledge may not necessarily translate into practice as has been previously reported by Lartey [24]. Thus, interventions should not only concentrate on educating the mothers to improve their knowledge but they should also support them to overcome barriers that may be preventing them from utilizing GMP services. Nonetheless, future studies should explore the reasons why mothers do not utilize GMP services although they have high knowledge.

The current study is not without limitations. Its cross-sectional nature makes it difficult to establish causality. The findings may also be liable to recall and social desirability biases. In addition, institution-based nature of the study in which we recruited only participants that attended the monthly welfare clinic, may affect the generalizability of our findings to non-attendees as these group of participants may have different characteristics compared to those attending with respect to the outcomes of interest. Notwithstanding the limitations, the study has important strengths. The findings add to the literature regarding our understanding of the utilization of GMP and its associated factors in Ghana and other countries having similar development trajectory like Ghana. It makes available evidence that may be useful for the design of interventions to improve GMP attendance and reduce child malnutrition.

## Conclusion

Utilization of GMP services was low and was much lower in children aged 24–59 months. High knowledge regarding GMP was not a determinant of utilization of GMP services. There is a need for healthcare providers to continue to encourage and support mothers to continue to bring their children for GMP services till the children turn 59 months.

## Data Availability

Data is available upon request from the corresponding author.

## Abbreviations

CWC: Child Welfare Clinic
CHPS: Community based Health Planning and Services
FAO: Food and Agriculture Organization
GMP: Growth Monitoring and Promotion
GHS: Ghana Health Service
GDHS: Ghana Demographic and Health Survey
GSS: Ghana Statistical Service
IYCF: Infant and Young Child Feeding
MOH: Ministry of Health
NSD: Nanumba South District
SPSS: Statistical Package for Social Sciences
UNICEF: United Nations Children’s Fund
WHO: World Health Organization
WIFA: Women in Fertility Age
